# A guide to setting up and managing a lab at a research-intensive institution

**DOI:** 10.1186/s12919-021-00214-7

**Published:** 2021-06-22

**Authors:** Bob Goldstein, Prachee Avasthi

**Affiliations:** 1 Biology Department and Lineberger Comprehensive Cancer Center, The University of North Carolina at Chapel Hill, Chapel Hill, NC 27599 USA; 2 Department of Anatomy and Cell Biology, University of Kansas Medical Center, Kansas City, KS 66103 USA; 3 Department of Ophthalmology, University of Kansas Medical Center, Kansas City, KS 66103 USA; 4 Department of Biochemistry and Cell Biology, The Geisel School of Medicine at Dartmouth, Hanover, NH 03755 USA

**Keywords:** Postdocs, Faculty, Laboratory, Career development

## Abstract

Postdocs who land faculty jobs at research-intensive institutions need to juggle several new large-scale tasks: identifying space and equipment needs for their lab, negotiating the hiring package, outfitting the lab with supplies, building a team, and learning to manage time in ways that can promote productivity and happiness. Here we share tips to help new hires think clearly about each of these tasks.

## Introduction

You’re on the job market, and you’ve just been offered a job. Congratulations! Now brace yourself, because the next steps will involve some quick decision making. You’ll be asked for a start-up list detailing the costs of equipment and supplies that you’ll need to set up your lab, and you’ll be negotiating important job details — all while thinking about how you’ll build a team, a career, and a life as an independent scientist.

This short guide covers some advice that we’ve shared with postdocs who had just been offered faculty positions at research-intensive institutions, or to postdocs who were thinking ahead to a time when this may happen. Here, we cover how to set up and manage a lab. (A separate article in this series covers steps that follow toward tenure [1]). We have prepared this written guide to contribute to leveling the playing field for newly hired faculty, by demystifying some key steps to success that we learned little-by-little from various sources.

This guide is not exhaustive. Rather, it seeks to raise some points that can be useful to consider at this stage. We recommend using this guide, together with other sources [2–18] and those referenced throughout this guide, as starting places for discussions with peers to brainstorm ideas for setting up your own lab. Discussions with peers facing similar challenges can be invaluable for your own success and to help you face the challenges with some optimism that you can succeed. For this reason, we recommend forming networks of peers who face similar issues. The possible avenues for forming such networks may come naturally to some. You may want to ask peers locally to gather on occasion for informal meetings; use conferences in your field to get together with groups of people who are not well represented at your own institution; and use communities like Future PI Slack [19] and New PI Slack [20] to communicate about problems you’re facing, to seek solutions, and to create new channels within those platforms for building needed spaces. However, it is not unusual for many new PIs to feel like outsiders because of issues they face that some of their peers do not. Videoconferencing tools and messaging platforms like Slack make it easier than before to initiate groups who share related challenges. A little work on cultivating the community you need if it doesn’t already exist can go a long way, and if a gap exists in the support landscape for you, it likely does for others as well. This shared need is what binds a nascent group together. People are in general happy to meet informally to learn from each other.

An important point to keep in mind: As a newly hired PI, you can make mistakes, and you can be sure that you will. We all do. It is common for early-stage principal investigators (PIs) to regret some of the decisions they make regarding hires or equipment purchases. Many early-stage PIs repeatedly and subtly change their approaches on exactly how to interact productively with their lab members. There are many paths to success. Some trial and error to finding your own path is inevitable. Rather than expecting perfection, it can be healthy to view some trial and error as valuable in your learning process.

## Identifying space and equipment needs

One note before we get to space and equipment needs: Back when you were initially interviewing for jobs, each interview was really a two-way interview – they were considering you for the job, and you were considering them as colleagues and their institution as a place to settle into. But in general, initial interviews are biased heavily in one of those directions: in most searches, they are generally interviewing their top five or so candidates from hundreds of applicants for just one job. As a result, it’s generally wise to try to enjoy the interviews by focusing on your shared interests in science, not money, and to avoid saying anything that could be perceived as a demand at this stage. It’s a good idea to ask relevant questions, for example about availability of graduate students and support for graduate students during rotations, but it’s generally not a good time for expressing expectations about start-up or salary totals (which we discuss more below). If your needs are typical, then saying so is a useful answer to any questions you’re asked at this stage about your start-up needs – to settle a chair’s worry that you’ll have atypical needs that can’t possibly be met, for example. Then pivot right back to your shared scientific interests.

Once you have a verbal job offer in hand, things change. You’ll plan a second visit to see the lab space and to think concretely about your equipment needs. You’ll build your start-up list, which will commonly be the basis for negotiations with a department chair (or sometimes a dean) before you accept the job offer. If you and your partner are both seeking jobs, this is when you’ll raise that. Remember that you are negotiating with a future colleague, so always negotiate in good faith. With any communication about your start-up needs, you might like to also re-express enthusiasm about the job and gratitude to the chair for helping to work out the details. It’s important to find a good balance where you are clear about any needs to accomplish what they’re hiring you for, while also starting off on friendly and professional terms with your future colleague. In general, the department chair is on your team: the chair will want new hires to be set up well to succeed. Be clear that you’re enthusiastic and that you just want to make sure you will be set up for a strong start there. Work to arm the chair with relevant information, including any other offers you have, to help them justify start-up costs to a dean, provost, or other institution sources.

You can envision your space needs as one bench and one desk per person, plus common space for equipment, shared resources, and other needs, and some room for growth including temporary summer students and rotation students. Looking at the benches and common spaces in your postdoctoral lab will be a useful guide, but be sure to consider all of the spaces, for example the spaces used for incubators, freezers, microscopes, tissue culture, and any equipment that’s outside of the lab in shared equipment rooms. If you want to really make sure the space will be adequate, consider the footprints of any atypical equipment or setup you’ll use. For example, this could include space for maintenance of your research organisms, microscopes, or custom-built rigs for specialty applications. Space discussions after accepting the job can be contentious, so this is a good time to ensure that you’ll be comfortable with your lab’s space.

Interviews can be an opportunity to ask to see the space, if a specific space is designated. Just as with meeting potential future colleagues, seeing a space can give you a gut feeling about a place that can contribute to your decision about whether to accept an offer. Some PIs like their office connected to the lab, where they can interact with lab members and catch any misconceptions in conversations. Others prefer a space that is separated from the lab for quiet writing and thinking or for more frequent interactions with other PIs. Nearby labs can affect a lab’s atmosphere significantly too. Consider the value of having colleagues nearby with whom you anticipate camaraderie, as well as potential collaborators and PIs who might serve as natural mentors to you. Physical proximity can contribute to productive collaboration [21]. Even a reasonably short distance can be the difference between interacting with colleagues and not. Note that in some institutions, and particularly in some medical schools, space is linked to funding using a specific space formula in which square feet of lab space is determined for each lab by how much grant funding they are bringing in. After you have a job offer, it is a good idea to ask the chair if a space formula is used, and how frequently it is calculated (i.e., will you lose lab space if you have a brief gap in funding, and conversely how quickly can your space grow if your grant funding grows).

## Building and negotiating the start-up package

Once you’ve landed a job offer and you’ve replied that you’re interested in the offer, you’ll likely be asked for your start-up list. It’s a good idea to at least get a start on putting one together in advance. The start-up list is where you will be clear about your itemized needs to set up your lab. If you are being recruited in part for a specific skill, and they don’t have the infrastructure you need, it is important to say so. You should be clear about things that are absolutely required to bring the strengths they want. You sold them on your strengths in your job talk(s), so your start-up needs should not be a surprise to anyone. Indeed, sometimes recruitment is the department’s chance to justify investing in expensive equipment that they’ve been wanting.

Your start-up list is often the best opportunity you will have to secure equipment that is necessary for your research but that is too expensive to be paid for from most grants. In rare cases of an especially expensive piece of equipment, you might propose that it be shared to help make the case for utility to the department, but in general you will want full access to your own lab’s equipment instead. For such large pieces of equipment, consider whether your usage will be enough to justify covering maintenance costs from your grants and/or efforts by you and your lab for maintenance, or whether some equipment might be better to propose as a shared departmental resource in an existing core facility. These different models each come with their own strengths and drawbacks. For instance, in core facilities, usually you will need to pay by the hour for use of equipment, and your lab’s access may be limited, but the equipment will be maintained by someone else in a core space.

Rather than reinventing the wheel by building a start-up list from scratch, most people collect multiple start-up lists from peers and/or communities like Future PI Slack [19] and use those as examples as they build their own list. Just as with envisioning your space needs, it can be useful to take stock of what you’re using in your postdoc that you’ll need in your own lab, along with things you’d like to upgrade, as technology advances quickly. If you ask, companies will bring expensive equipment like microscopes to you to try out (such a demonstration of equipment is referred to as a “demo”), which can allow you to compare equipment and support from different companies. As you build your start-up list, look back at your application materials. What did you say your research goals would be? Are you requesting what you’ll need to pursue those goals?

The amount of money that is reasonable to expect for start-up varies tremendously by type of job and institution. It can be helpful to talk to colleagues at peer institutions or to use communities like Future PI Slack [19] to get an idea for what the market is offering. After initial startup costs, personnel are often the biggest cost. Most people will request 1–3 years of personnel costs to cover the cost of initial personnel until the first grant is awarded, for example for a technician and your first 1–2 graduate students. At some institutions, you will also be covering part of your own salary from start-up (and later from grants). For all personnel, remember to include fringe benefit costs as well as salary, or tuition for students if you are expected to cover this. You can contact your future department’s accountants to ask for useful information on the standard fringe costs for grad students, postdocs, and technicians. Certain potential costs in your lab may be covered by other sources in the long term, for example when graduate students land 1–2 year spots on training grants, but be sure to gauge the likelihood that this will happen on average, for example by asking for the success rates of graduate students applying for training grant spots. If you are choosing between multiple verbal offers, be wary of comparing start-up totals when deciding which offer to accept. What is included in start-up totals can vary between institutions, and being somewhere you can succeed in your goals and enjoy your colleagues will be far more important in the long run.

Once you and the chair iron out details, you should receive an official offer letter. At this stage, it is wise to find some new and experienced PIs whom you trust (for example, PIs at your postdoc institution) to check over your offer letter. They can help spot important gaps, and they can help build your comfort with negotiating as needed. Getting such input at this stage can be invaluable, because the offer letter is generally treated like a contract by institutions, and yet initial offer letters are often missing some critical details. As a result, it is not unusual to tactfully ask the chair for a revised offer letter that details all important points and expectations, including the precise teaching load, any restrictions on when and how your start-up total is spent, what space the lab will occupy and well-defined arrangements for any shared space, and specific plans laid out in case the space is not fully ready when you start, with everything (including gas lines and air lines) working. Spaces that require significant renovations come with a potential drawback: it is not unusual for schedules to stretch out for longer than planned because of unforeseen issues either with the space or with other jobs affecting engineering and facilities’ workers schedules. It is helpful to also have a specific statement that any needed infrastructure repairs or renovations will be completed before the lab opens, with contingency plans if that cannot happen, and that lab furniture (lab benches and chairs) will not be charged to your start-up allocation. Most institutions will give faculty two pre-tenure semesters of relief from teaching, to help with getting a research program off the ground. You will want to be strategic about when you use these (it can be helpful for starting the lab to have one semester away from teaching as you begin, and you might delay the other semester if possible until closer to tenure, although different PIs have different preferences). You also might consider in your teaching assignment whether you can teach courses that first year graduate students take, to give you exposure to potential rotation students. Be sure that your start date is specified and understand what the process would be if there were a need to adjust it, and by how much. Be sure that your chair has explained to you how your start date will play into your promotions and tenure timeline, your teaching roles, and your recruitment of graduate students.

If you are enthusiastic about the written job offer but you have any remaining major unmet needs that seem reasonable, then consider picking one major need and requesting that. For example, if more than one lab space is available, and one option is near colleagues that you dream of being near, it would be fine to tell the chair this. If some important details are still omitted from an initial or revised offer letter and yet you’re keen to accept the offer, it can help to email a conditional acceptance stating your own understanding of some specific unwritten details, so that a chair can correct any misconceptions at this stage, and so that you will have some record of verbal understandings to refer to in case the department chair changes.

With all of your negotiations, remember again that your chair will want you to succeed; they’re expending significant effort and significant resources for your benefit; and you’re beginning a long-term professional relationship. With these things in mind, while you need to be clear during any needed negotiations, you’ll also want to be tactful, and maintain a friendly, professional tone.

Once you’ve formally accepted the job, celebrate!

## Opening the lab, onboarding new lab members, and building a team

After celebrating, you’ll have a chance to get wheels in motion for selected things that will benefit from some early action. Two things are useful to consider tackling first: (1) So that incoming graduate students and undergraduate students will see your new lab as a possibility, get yourself added to your new department’s and/or graduate program’s website; build your own lab’s website; and use social media to announce your lab. (2) Request demos of large pieces of equipment, like microscopes, from companies. Companies can generally offer to demo equipment on site at your postdoc lab. If you make decisions on big pieces of equipment early, this accommodates lead time often necessary to get the equipment delivered to your new lab so that your first rotation students can start in the lab without too much delay. Large pieces of equipment can sometimes have specific parts on backorder, and any resulting delays can create problems. For example, the microscope you need will feel like a useless doorstop until the most critical lens arrives. Some institutions will allow you to spend some start-up funds before you arrive, so that you can begin to place orders early. If support from equipment company representatives is important after purchase, as it can be for microscopes (a rep that can loan parts that need repair can save a lot of headaches), then be sure to ask your future colleagues about the effectiveness of reps for different companies before making major purchasing decisions.

Sometimes another lab is closing down or moving and leaving glassware, pipettors, and equipment behind. Once you’ve accepted a job offer, it’s a good idea to ask the chair if this is anticipated, and plan to scavenge useful items with permission when they become available.

Before you arrive in your new position, you might consider hiring a short-term personnel to start when you do, or even earlier, to help with purchasing and unpacking. In general, hiring people is governed by more defined rules than is buying materials, because hiring is regulated by employment laws. These laws can require openly posting positions, which can help you get a larger and more diverse set of applicants than you might otherwise. You can further grow and diversify the applicant pool by encouraging people to apply, soliciting recommendations for such people from other new PIs through New PI Slack [20], Twitter, and from colleagues, especially junior faculty at your new institution. You may also consider recent graduates or gap year students. You can also hire work-study students at your new institution to help with setting up the lab. Your new department’s human resources specialists can advise you on navigating hiring requirements.

Equipment and supply purchases are sometimes governed by contracts negotiated between vendors and the university. Your department’s accountants can explain any equipment and supply purchase rules and whether you are required to complete any training to make purchases. Product representatives from multiple suppliers may reach out to you as new PIs often make many purchases at once. New investigators can sometimes negotiate discounts. While not all of the lab startup deals from suppliers are in fact a deal, some significant discounts are possible when buying in bulk. Ask other labs what they pay for frequently-used services like DNA sequencing and for ordering primers so that you can be aware of discounts to request from your sales reps. Some institutions allow purchases from Amazon, or eBay for used equipment. As you begin purchasing equipment and supplies for the lab, you will spend some time trying to cut costs on big purchases and frequent purchases. It will take some practice to minimize the time you spend researching economical purchases. At some point, you may delegate the supplies purchasing role to a technician or assistant that you hire, or have each person in the lab make their own supplies purchases as needed, but it will be useful for you to learn the ropes at first so that you can be aware of any unanticipated issues.

Some newly hired PIs will get a start on writing their first grant proposal, or even submit one, before arriving. Grant writing strategies are covered in a separate article [1]; here, we review just selected matters that may help with envisioning the process in advance. Once you have a suitable funding source (like NIH) identified, it can be helpful to email a program officer there that handles grants in your subfield to ask for a short phone or video meeting. Program officers can evaluate whether the aims you plan to propose are suitable for a specific funding mechanism or review panel, and sometimes they will share other advice. You can also start to collect a list of awards specific to junior faculty such as Pew and Searle Scholars programs and ask your new chair about being nominated for ones that only accept limited numbers of proposals from each institution; some applicants will ask about being nominated for these before accepting a job offer. Some institutions keep especially useful resources of funding opportunities that anyone can access online, including funding opportunities reserved for early-career faculty and underrepresented minority researchers [22, 23]. Other junior faculty may share lists of grants they have applied for, and they may share successful proposals to use as models as you prepare your own. For all grant proposals that you plan to write, be sure to contact your new institution’s office that handles grant submission (commonly called a Sponsored Projects office or a Sponsored Research office) as well as the administrative people handling grant proposals in your department to find out what they will need. Within-institution deadlines are commonly a few days to a week before a funding agency’s deadline, because they will need to check proposal paperwork for problems and sign off on proposals. Administrative people within your own department can sometimes help with preparing budgets and other paperwork parts of proposals; it’s a good idea to ask faculty in your new department about the help that department administrators can typically provide.

Upon arriving in your new position, you’ll start to build a team. For many new PIs, building a strong team can become one of their most satisfying new roles in science. Building a strong team can also be challenging, and it can be critical to your success as a PI. You will want to consider the extent to which you will micromanage lab members’ work and/or encourage and support independence, and for people to work independently vs. in teams, to promote both your scientific goals and your lab members’ career development. Even in labs where each lab member has a completely independent project, the group of people has the potential to interact in healthy ways that can help everyone. People may have skills and interests that complement each other (like the old Super Friends cartoons — Wonder Woman stops the bullets, the Wonder Twins transform into useful forms, and Aquaman takes care of underwater tasks, or teaches others how to do so). It can be helpful to seek lab members with these criteria in mind. Diversity in multiple senses can help contribute to a strong team [24, 25]. For this reason, it is important to fight the urge to look for clones of yourself in prospective hires. And it can be helpful to consider your own goals, as you framed them in your job talk(s) or in grant proposals that you are envisioning. Sometimes lab members with specific interests can help relieve demands on your time; for example, a lab member interested in microscopy might be happy to serve as a second point person for interacting with microscope companies.

As you’re considering taking on new lab members, keep in mind whether potential lab members have emotional maturity too. A strong experimentalist who cannot interact well with a diverse team and respect others (often described as a “dominant negative” lab member) can harm team dynamics and/or slow other people’s research progress. Interviews and rotations can be an opportunity to look for healthy interactions between people. There may be a temptation to avoid conflict or hang on too long to a poor fit, but all lab members contribute to the collective environment, and a PI’s inaction often can be damaging. And a group with healthy interactions will contribute strongly to their *own* team building atmosphere. It can be helpful to watch for natural team-building initiative among lab members and to encourage it.

You’ll want to make your lab a welcoming place for potential lab members both in terms of the atmosphere among lab members and in terms of how the lab space is organized. As your salary will generally jump up in the transition from postdoc to PI, it may be helpful to consider the last $1000 or so of your annual PI salary as for the lab — for a lab coffee machine, bluetooth speakers, a food fridge, and in the longer term, for lab dinners and other social events. You’ll thank yourself later! Also remember that such perks are not a substitute for the culture you’ll seek to build: it’s even more important for lab members to feel nurtured and supported. If you’re naturally shy, develop regular habits to be in the lab interacting with people. In general, people in the lab will appreciate when their opinions are solicited and considered, for example when you are considering adding new lab members or making major purchases. You may not always agree with the feedback you get. You may even overrule the feedback for important reasons, but listening and understanding the perspectives of those who will be interacting most frequently with new people and equipment is invaluable [26, 27].

It’s wise to give yourself plenty of chances to interact with potential rotation students: interview as many prospective grad students visiting your department or graduate program each spring as possible, seek opportunities to meet the new grad students who arrive each fall. In your first couple of years, consider volunteering for the admissions committee that reviews graduate school applications for your program. Become aware, though, of the time you would be committing first. It’s a good idea to try to limit your committee work before you have your first grant supporting the lab’s research (and chairs and colleagues will generally respect this need, assigning you to a light load of select committees at first), but the admissions committee can be a valuable one to join when you’re seeking graduate students if the time required is not too burdensome at your institution. For campuses with large numbers of partially overlapping departments and programs, you might also explore whether you can be affiliated with multiple graduate programs on your campus, to recruit from multiple pools of incoming graduate students each year. Having access to multiple pools of graduate students may aid in your goal to find graduate students who are good fits for your lab.

The graduate students who start in your first year or two will have incentives well aligned with your own goals toward tenure: making discoveries and reporting them in publications in your pre-tenure years (hence the advice above about setting yourself up to take rotation students into a fully functioning lab as early as possible). In general, when your own incentives and the incentives of the people in your lab are well aligned, this can help many things in your lab go smoothly. For this reason, and because you’ll be working toward healthy relationships with the people in your new lab, it’s a good idea to get to know what motivates the people in your lab both as you’re considering taking people on and as their goals evolve over time [28]. Do they have enough freedom to encourage their own creative thinking and for them to grow as independent scientists? Having an atmosphere where people feel comfortable taking some risks and also talking about their motivations can help. It is especially important to consider the challenges and circumstances of those who may come from a different background than you. You can encourage lab members to complete any of several types of individual developmental plans, for example MyIDP [29], at least annually, as an opportunity to review their long-term career goals and to review whether they’re working toward those goals. Graduate students and postdocs have diverse career interests and skills they’re seeking to develop [30–33]. They will need to spend some of their time exploring options for their continued careers, for example by attending workshops, gaining teaching experience and experience in scientific outreach. Your support toward their own goals will be appreciated. Often, time spent cultivating one’s soft skills, career path, and network will energize, refocus, and motivate their science as well [34].

Recruiting postdocs when you’re a junior faculty member can be difficult because you will be less well known than many of your senior colleagues. Some new PIs work on developing an online presence, for example on Twitter, to help students and colleagues become aware of their work. Twitter can be a great place to share what you do, solicit feedback, and work on community building. Opportunities to interact with graduate students at other institutions — for example giving invited seminars, contributing a little to courses that students travel to, or meeting graduate students at conferences — can also contribute to recruitment of postdocs to your lab. These opportunities also serve the reason that most of us do science in the first place, i.e. to make discoveries and to share the discoveries with each other. Sometimes these opportunities can seem hard to come by as a new faculty member, but consider adding your name to lists of seminar speakers at New PI Slack or other initiatives that serve to increase speaking opportunities for early career or underrepresented groups. Once you have postdoc applicants, it’s a good idea to talk to their PhD advisor directly, because conversations often produce more candid evaluations of strengths and weaknesses than a letter will.

Undergraduate students can also contribute to a lab environment. Training undergraduates has the potential to contribute to both your graduate students’ and postdocs’ career development. From large undergraduate student populations, there is a great potential to recruit ethnically and racially diverse undergraduates, who can contribute to strengthening your lab and the scientific field more generally by bringing diverse perspectives. Use your colleagues’ input on how to recruit undergraduate researchers at your institution and how to structure the undergraduate research experience to support benefits to both the student and the lab’s research goals.

These days, PIs often generate an onboarding document that outlines lab policies and expectations — what you expect from lab members, and what they can expect from you [35, 36]. These documents can save time and reduce miscommunication. It is helpful to spend considerable reflective time envisioning the type of lab dynamics and culture that you want to build. Create a prioritized list of the character traits, work ethics, diversity, and professional behaviors that you envision in your group, considering expert advice [9, 24, 25], and use these to build a mission statement for the document. Once you have members in the lab, it can be useful to rebuild this document on occasion with everyone’s input, to help make the document more useful and to increase everyone’s genuine support for a useful set of expectations. As you gain lab members, you will likely want to improve your ability as a mentor; online resources can help [37–39], and some institutions offer local training workshops.

Maintain a professional relationship with your lab members. Being a new PI can be lonely at times, and it can be tempting to put yourself on equal footing as one of the group. But keep in mind that there is an inevitable power dynamic: they will generally be aware that you have a great deal of control over their ability to succeed [40, 41]. Ignoring the existing power dynamics can lead to issues down the road. To promote a healthy environment, it can be useful to encourage lab members to cultivate broad networks of peers and additional PIs to give them input, to acknowledge that you and members of the lab may have interpersonal conflicts at times, and to start off relationships stating that you are committed to working through any conflicts that do arise.

In a position now where you have some responsibility for others (mentoring, teaching, serving on admissions committees, etc.), you should consider with some care not just what you’re doing but also what you’re *not* doing. In positions of responsibility, people are reasonably held responsible for both, which can be an equally daunting and exciting challenge to aim to meet. Take some time to read and/or discuss with colleagues issues that are important to the practice of science — for example, diversity issues and inclusive environments, unconscious bias, mental health issues, and ethical conduct.

There are many ways to be a successful independent scientist. For example, some PIs choose to work at the bench their entire career with a small number of PhD students. Some prefer challenging themselves to run effectively as big a lab as possible. Some prefer a lab of mostly postdocs, or mostly PhD students, or undergraduate students. Some choose to focus on developing new techniques for a field. Some keep research topics continuous from each lab member to the next, and others prefer to try new lines of research frequently. Keep in mind that you don’t need to be all of these things. Your colleagues in your institution and your field provide you a variety of useful models to consider as you shape your own ideas of the kind of scientist you’d like to be.

## Managing your time for productivity and happiness

You’ve made it to a highly sought-after position, so you’ll thank yourself later for some thinking at this stage about how you’ll develop your career in a form that will contribute to your long-term well being.

Every new PI establishes relationships with many people beyond their own lab members. Among these people, other junior faculty can serve as an important peer support group, because you’ll be going through some experiences in common. It can be helpful to schedule informal gatherings with this group, as well as gatherings focusing on topics like teaching or specific scientific topics. Many new PIs are surprised to learn that some thorny issues they face have already been faced by other new PIs. The solutions that your peers have found for their own productivity and happiness can be useful to consider. It can also be valuable to cultivate relationships with people who can serve as informal mentors, i.e. more senior faculty who share your vision for how to run a lab, and who can provide input on your grants and manuscripts (and when you do share grants and manuscripts, it’s courteous to let people know a few weeks in advance the specific date when you’ll share something, and to ask if they can return comments within a week, so that they can schedule in advance some time for reading and commenting). If your department does not have a formal process for existing PIs to mentor new PIs, you can ask your chair to recommend some informal mentors. Also consider mentors from outside of your own institution who might agree to an occasional meeting by video or at conferences. As you collect wisdom from peers and mentors, consider practices they use in common as well as input from the outliers whom you admire. Remember that there are many paths to success and almost as many ways to do the job as there are people doing it [42]. For this reason, it’s important to decide what you value, and keep an eye toward liking the person you’ll become to get tenure and the job that you’ll have at that stage. It is important to stay true to your values rather than solely ensuring that you meet expectations required to keep and advance your career.

You will also establish new relationships with administrative staff members of your department — accountants, human resources specialists, facilities maintenance people, and others. Be sure to treat administrative staff members respectfully, and be friendly. Some PIs make the mistake of finding themselves in a position of some authority for the first time and, in the interest of time efficiency, losing a little of their naturally friendly and human demeanor when interacting with staff members. You will likely know these people for a long time. Administrative staff can be tremendously effective at navigating quirks of institutional rules, so remember that you stand to learn a lot from them.

Your own time management is important. Your research effort and time allocation will be completely unlike what it was as a postdoc. At first, you are, in a sense, your own best postdoc, so it can be wise to continue bench work yourself at least until lab members are well trained. In the long run, your time management will likely involve some trial and error in figuring out what works best for you, as well as trying out new habits now and then throughout your career. The pace of work may feel ramped up at first, given your role in multiple projects in the lab at the same time. This change typically involves an attitude and skills adjustment to manage more projects. You’ll need to be strategic about your research goals, not spreading yourself too thin across too many distantly related projects, and you’ll need to find your own best balance for effort toward research, teaching, and other roles. Teaching a course for the first time generally involves a bigger time commitment than new faculty anticipate, both for preparing the material to be taught and for learning how to manage a course effectively and efficiently. You’ll want to do a job you’re proud of in research, teaching, and your other roles, but remember that in the end you will need to find a balance that you can be comfortable with. If it takes you longer than you expect for setting up your lab and beginning to teach, give yourself a break by reminding yourself that this is a common experience for new PIs. You’ll likely improve at juggling multiple roles over time. Help is available for faculty looking to improve their abilities to do so [14].

One key issue of time management is how to respond to others’ expectations of your contributions in your department or in the field. It will be important to be a good citizen by doing your share of work in your department and in your field of science, but you’ll also need to learn to say “no” in order to avoid overextending yourself. For example, for reviewing papers and grants, a good rule of thumb for being a good citizen is to only review those that are within your own field and to review roughly as many grants and manuscripts as one’s own lab’s work incurs (for example, plan to review roughly 2–3 manuscripts per manuscript that you submit for peer review). It’s always okay to respond to requests with a polite, “I’m overbooked with other commitments and wouldn’t be able to take this on” whenever this is the case. A good option when turning down a request is to recommend a colleague or trainee that could use the opportunity. This makes it easier to say no, solves a problem for the requester, and helps others.

When deciding if you are indeed overbooked with other commitments, be sure to reserve time both for things that are urgent and things that are important but not urgent. Important but not urgent tasks are often key to long-term success, but are also the easiest to postpone. These tasks will be challenging to complete unless you specifically set aside time in your schedule. For example, grant proposals become increasingly urgent as a deadline approaches, and beginning early to set out a plan and stick with it may require you to mark out planned milestones month-to-month on your calendar. Commonly used grant application guides can help with planning out a useful schedule for yourself [43]**.** For important tasks, it is also common to fill up all available time with a specific task. This may result in spending a week to prepare a lecture or complete a first draft of a piece of writing that could have been done in a focused day. A useful method of dealing with this phenomenon is to allocate dedicated time, and no more, in one’s schedule for a specific activity (sometimes called “timeboxing”). The increased focus can reduce anxiety when sufficient time is protected for an activity. This practice can also reduce the amount of overall time spent, thereby increasing productivity. There is no shortage of management hacks taken from the business world that can benefit a new PI balancing many productivity, hiring, leadership, and organizational challenges for the first time. We are often not trained for many of the new roles that a PI takes on, but existing resources can help [6, 8, 9, 44, 45].

Another trick for making good use of your time is to double up on use of commitments when possible. Consider whether certain of your commitments can double as useful topics for lab meetings, or training opportunities for grad students and postdocs. For example, some journals will permit reviewing a manuscript together with a member of your lab. Doing so can satisfy multiple of your goals — contributing to your good citizenship in the field, keeping you and a lab member discussing ongoing research in your field, and serving as a valuable training experience for the lab member.

Keep in mind that some opportunities may not have a clear proximal benefit but may increase your exposure in the community and with your colleagues, and/or allow you to get to know colleagues you admire. Your reputation as a scientist is one criterion for tenure, as reflected by letters that your tenure committee will solicit from colleagues of your choosing and of their choosing. This reputation will be based in large part on what you discover and publish. For this reason, it can feel especially satisfying to say yes to opportunities that increase your exposure in the field specifically when you have discoveries that you’re especially proud to share; in this way, you can bring something of value to others, for example in invited talks you give, and you can gain input on your work in the process. There is a firehose of research out there, and it is not necessarily anyone’s job to pay particular attention to your or your lab members’ discoveries. It is important to appropriately credit your lab members for their discoveries in talks and on social media, and colleagues in your field will appreciate learning about these discoveries.

Lastly, don’t forget to do what you need beyond your job – time with family and friends, and doing other things you enjoy! You can expect to experience some failure and resulting stress at work, along with some successes, and overworking can be emotionally draining. If you’re the kind of person who feels guilty taking time away from work, you can comfort yourself by remembering that you’ll likely be a more creative scientist and a better mentor if you are taking time to do what you enjoy and what’s important to you outside of work.

## Data Availability

Not applicable.

## Abbreviation

PI: Principal investigator

## References

[1] Boyce M, Aguilera R. Preparing for tenure at a tesearch-intensive university. BMC Proc. 2021;15(2). 10.1186/s12919-021-00221-8 PMC8217977 34158043

[2] Making the Right Moves: A Practical Guide to Scientific Management for Postdocs and New Faculty. https://www.hhmi.org/science-education/programs/making-right-moves. Accessed 7 Jul 2020.

[3] Barker K (2010). At the helm: leading your laboratory.

[4] Noor MAF (2012). You’re hired! Now what?: a guide for New science faculty.

[5] Jahren H (2016). Lab girl.

[6] Newport C (2016). Deep work : rules for focused success in a distracted world.

[7] Cohen CM (2005). Cohen SL.

[8] Bungay SM (2016). The coaching habit.

[9] Coyle D (2018). The culture code: the secrets of highly successful groups.

[10] Somerville LH, Cunningham WA, Gruber J, Van Bavel JJ, Lewis NA (2019). Three keys to launching your own lab.

[11] Petry S. Learning the art of leading a lab. ASCB. 2017; https://www.ascb.org/careers/learning-art-leading-lab/. Accessed 7 Jul 2020.

[12] EMBO Lab Leadership Courses. https://lab-management.embo.org/. Accessed 7 Jul 2020.

[13] National Institute of General Medical Sciences (2020). Starting Your Own Lab with Dr. Olivia S. Rissland and Dr. Prachee Avasthi.

[14] Faculty Diversity: On-demand access to the mentoring, tools, and support you need to be successful in the Academy. https://www.facultydiversity.org/. Accessed 15 Jul 2020.

[15] Career development courses, workshops, and programs from ASCB. https://www.ascb.org/career-development/. Accessed 15 Jul 2020.

[16] Careers in Biology. iBiology. https://www.ibiology.org/careers-in-biology/. Accessed 21 Jul 2020.

[17] SACNAS Webinars. https://www.sacnas.org/what-we-do/webinars/. Accessed 18 Sep 2020.

[18] SACNAS Professional Programs. https://www.sacnas.org/what-we-do/professional-programs/. Accessed 18 Sep 2020.

[19] Future PI Slack. https://futurepislack.wordpress.com/. Accessed 7 Jul 2020.

[20] New PI Slack. https://newpislack.wordpress.com/. Accessed 7 Jul 2020.

[21] Claudel M, Massaro E, Santi P, Murray F, Ratti C (2017). An exploration of collaborative scientific production at MIT through spatial organization and institutional affiliation. PLoS One.

[22] Opportunities F (2016). Vice provost for research at Johns Hopkins University.

[23] Funding Opportunities for Women and Minorities, UC Berkeley Sponsored Projects Office. https://spo.berkeley.edu/fund/womenminority.html. Accessed 21 Jul 2020.

[24] Rock D, Grant H (2016). Why Diverse Teams Are Smarter.

[25] Taylor TA (2018). Diversity, Inclusion and Culture. How to Build Great Teams.

[26] Scott K (2019). Radical candor: fully revised & updated edition: be a kick-ass boss without losing your humanity.

[27] Stone D, Heen S (2014). Thanks for the feedback: the science and art of receiving feedback well (even when it is off base, unfair, poorly delivered, and frankly, You’re not in the mood).

[28] Teater B. 7 qualities to look for in a scientific mentor. The Jackson Laboratory https://www.jax.org/news-and-insights/2015/october/seven-qualities-to-look-for-in-a-scientific-mentor. Accessed 7 Jul 2020.

[29] myIDP. http://myidp.sciencecareers.org/. Accessed 8 Jul 2020.

[30] National Postdoctoral Association (2009). NPA core competencies.

[31] Tracking Career Outcomes at Institutions (2018). Future of Research.

[32] Postdocs’ Guide to Gaining Independence. https://www.niaid.nih.gov/grants-contracts/postdoc-guide. Accessed 7 Jul 2020.

[33] iBiology Courses. https://courses.ibiology.org/courses. Accessed 7 Jul 2020.

[34] Avasthi P (2019). Synergy of science and career development | ASCB.

[35] Mehr S. Lab handbooks tweet thread: Twitter. https://twitter.com/samuelmehr/status/1139733291899080705. Accessed 8 Jul 2020

[36] Masters KS, Kreeger PK (2017). Ten simple rules for developing a mentor-mentee expectations document. PLoS Comput Biol.

[37] Office of Intramural Training & Education at the National Institutes of Health. https://www.training.nih.gov/. Accessed 12 Jul 2020.

[38] NIH OITE videos. https://www.youtube.com/channel/UCQQHo_QnuBxdfcsRy4INGGw. Accessed 12 Jul 2020.

[39] The Science of Effective Mentoring in STEMM. https://www.nationalacademies.org/our-work/the-science-of-effective-mentoring-in-stemm. Accessed 12 Jul 2020.

[40] Tintori SC (2018). Turning the microscope on power dynamics in the lab. Mol Biol Cell.

[41] National Academies of Sciences, Engineering, and Medicine, Policy and Global Affairs, Board on Higher Education and Workforce, Committee on Revitalizing Graduate STEM Education for the 21st Century (2018). Graduate STEM Education for the 21st Century.

[42] Heppert J, Heikes K, Bowie E, Siripurapu V. How cell biologists work column: ASCB. https://www.ascb.org/tag/how-cell-biologists-work/. Accessed 7 Jul 2020

[43] Workbooks - Grant Writers’ Seminars & Workshops. http://www.grantcentral.com/workbooks/. Accessed 7 Jul 2020.

[44] Smart G, Street R (2008). Who: solve your #1 problem. Ballantine Books.

[45] The National Center for Faculty Development & Diversity’s Faculty Success Program. https://www.facultydiversity.org/fsp-bootcamp. Accessed 15 Jul 2020.

